# Predictors of Attitudes Toward Autonomous Vehicles: The Roles of Age, Gender, Prior Knowledge, and Personality

**DOI:** 10.3389/fpsyg.2018.02589

**Published:** 2018-12-18

**Authors:** Neil Charness, Jong Sung Yoon, Dustin Souders, Cary Stothart, Courtney Yehnert

**Affiliations:** ^1^Department of Psychology, Florida State University, Tallahassee, FL, United States; ^2^Purdue Policy Research Institute, Center for Connected and Automated Transportation, Purdue University, West Lafayette, IN, United States; ^3^United States Army Research Institute for the Behavioral and Social Sciences, Fort Leavenworth, KS, United States

**Keywords:** autonomous vehicle, age, gender, personality, knowledge, attitudes, technology

## Abstract

Autonomous vehicles (AVs) hold considerable promise for maintaining aging adults’ mobility as they develop impairments in driving skill. Nonetheless, attitudes can be a significant barrier to adoption as has been shown for other technologies. We investigated how different introductions to AV, video with a driver in the front seat, the rear seat, and a written description, affected attitudes, as well as how individual difference variables such as age, gender, prior knowledge, and personality traits predict attitudes within a middle-aged (Median age = 34, IQR = 20, *n* = 441) Amazon Mechanical Turk sample. The 16-item attitude survey uncovered three factors: Concern with AV, Eagerness to Adopt AV technology, and Willingness to Relinquish Driving Control. ANOVAs showed that only age (younger less concerned) and gender, (females more concerned) were significant factors in Concern with AV. Only gender affected Willingness to Relinquish Driving Control, with males more willing. Multiple regressions that included previous knowledge level and personality traits showed a different pattern. Female gender and greater conscientiousness were associated with greater Concern about AV. Prior knowledge of AV was associated with less concern. Emotional stability and openness to experience were positive predictors of Eagerness to Adopt AV, whereas conscientiousness was a negative predictor. Prior knowledge and openness to experience, positively, and extraversion, negatively, were associated with being willing to relinquish driving control. These results suggest that different information dissemination campaigns are needed to persuade consumers to adopt AV technology. We discuss potential approaches.

## Introduction

Nearly 90% of all day trips in the United States are completed using a personal vehicle^1^. Given the emphasis on mobility with a personal vehicle, older adults’ quality of life in countries such as the United States having weak public transit systems depends on their ability to drive (Coughlin, 2001). Although today’s older adults are driving more miles than past ones (Insurance Institute for Highway Safety, 2015), most older adults eventually stop driving for themselves. Moreover, 20% of the global population will consist of older adults by the year 2050 (UN Department of Economic and Social Affairs, 2015). Thus, ways of keeping aging adults safely in the driver’s seat need to be identified.

Autonomous vehicles (AVs) potentially offer aging adults one way of staying in the driver’s seat as these vehicles would do most if not all of the driving (Reimer, 2014). A Pew study found that 75% of respondents anticipate the advent of AVs will help the elderly and disabled live more independent lives (Smith and Anderson, 2017). Benefits from AVs might also extend to caregivers, reducing their loss of income resulting from interruptions in employment due to the need to provide transportation to non-driving older adults (D’Ambrosio et al., 2012). Indeed, AV-related benefits to the United States economy have been estimated to approach $196 billion after accounting for increases in vehicle miles traveled, with this gain resulting from reductions of the number of crashes and the amount of time spent in congested traffic, as well as increases in parking savings (Fagnant and Kockelman, 2015).

While these projected AV-related benefits are substantial, consumer acceptance of AVs was recently cited as the AV’s “greatest constraint to growth” by United States Secretary of Transportation Elaine Chao (Hahm, 2018). Attitudes toward AVs seem to be multi-faceted, with drivers acknowledging the potential benefits of AVs while also expressing concerns (e.g., Howard and Dai, 2014). With respect to age, older adults are more hesitant about AVs than their younger counterparts (Missel, 2014; Payre et al., 2014; Duncan et al., 2015; Kyriakidis et al., 2015), but this gap is decreasing, with younger adults voicing more concerns than they had in the past (Abraham et al., 2018). With respect to gender, males have more positive attitudes toward AVs than females (Casley et al., 2013; Missel, 2014; Payre et al., 2014; Schoettle and Sivak, 2014, 2016; Kyriakidis et al., 2015), and this may be due to males generally having higher levels of sensation-seeking—a factor related to the intention to use AVs—than females (Payre et al., 2014). Additionally, more neurotic drivers are more concerned about data security in AVs than less neurotic drivers (Kyriakidis et al., 2015). However, the other Big Five personality factors as well as locus of control—the degree to which people believe they have control over the outcome of events in their lives—are not predictive of a driver’s intention to use AVs (Payre et al., 2014).

The way that AVs are introduced significantly affects attitudes. Nees (2016) presented Amazon Mechanical Turk (MTurk) workers with vignettes about a close friend or family member’s experience of AV ownership that were either idealized (e.g., the driver has little to no role when automation is active, and the automation successfully avoids collisions without the need for the driver’s intervention) or realistic (e.g., describes a balance of positive and negative experiences with the automation, the need for the driver to monitor the automation and intervene to avoid collisions), and found that those that received the idealistic vignette reported higher levels of AV acceptance. The possible loss of access to vehicle controls that might arise with higher levels of automation, has been found to be a source of significant concern (e.g., Schoettle and Sivak, 2014, 2016). Possibly having a fear of losing vehicle controls could be the result of the complete removal of the steering wheel and pedals, or because of the vehicle occupant’s position in the vehicle. However, there was no study that directly investigated the relationship between attitudes toward AVs and a perception of losing vehicle controls and how the possible relationship are associated with individual difference factors.

Thus, the current study had two main aims. The first was to assess whether an experimental manipulation affecting level of concern for losing vehicle controls would affect attitudes toward AVs in combination with the factors of age (younger versus middle-aged adults) and gender, and particularly whether these factors would interact. We tested these hypotheses with a between-subjects ANOVA on attitude factors (see below).

Our second aim, in light of previous studies that have relied on very brief, global assessments of attitudes toward AVs, was to assess the complexity of attitudes toward AV through factor analysis of a 15-item survey of attitudes toward AVs. We used the obtained factor structures as dependent variables for testing the hypotheses in the first aim. When we found no interaction effects in the ANOVAs, we also conducted exploratory multiple regression analyses to examine the linear effects of gender, age, prior knowledge, personality, and introduction type on attitude factors.

## Materials and Methods

### Participants and Procedure

We recruited 414 participants (United States residents only, age range = 18 – 73, median age = 34, mean age = 37.14, *SD* = 12.79; 160 males, 254 females) via MTurk. After they completed our Florida State University’s IRB-approved consent form (in accord with the Belmont report), we introduced participants to AVs by asking them either to: (1) view one of two video clips depicting an AV, or (2) read a description about an AV. Assignment to condition was randomized using a random number generator embedded in the task. Participants then completed a survey regarding their attitude toward AVs and then a survey regarding their personality traits. Participants then answered a few demographic-related questions, and were given a final item that tested attention to the experimental procedure. The script of procedures used in MTurk is available online on the Open Science Framework^2^.

Before agreeing to participate, we informed participants that we were interested in their attitudes toward AVs and that they would have an opportunity to withdraw from the study at any time without consequence. The study took approximitely 15 min to complete and participants received $0.25 for completing it.

### Materials

#### Description of AV

There were three different introductions to AV: (1) a written description of AV technology (Appendix A), (2) a video with a passenger in the front driver seat, and (3) a video with a passenger in the rear seat. The videos showed an AV driving on a highway in normal driving conditions. The videos also showed the inside of the AV; thus allowing participants to see the passenger’s location and how a cockpit works during automated driving. The rear-passenger video came from a laboratory developing various self-driving car technologies in South Korea and the front-passenger video came from a German car manufacturer. Both videos had no sound. The videos are available online on the Open Science Framework^3^. The written description provided information about AVs (e.g., definition of self-driving AV, features, how it works). Both videos and the written description took about 1.5 min to view/read.

#### Attidude Toward AV Survey

Participants first reported if they had ever heard of AVs before participating in the study. Participants then anwered 15 attitudinal items on AVs. Using a 10-point scale (1 = highly unlikely to 10 = highly likely), the first two attitudinal items estimated the likihood that participnats would ride as a passenger in a vehicle driven by either a taxi driver or automation. The remaining 13 attitudinal items measured participants’ attitude toward AVs (e.g., comfort or concern on self-driving vehicle, willingess to adopt self-driving vehicle) on a 5-point scale (1 = strongly agree to 5 = strongly disagree). All 16 items in the survey are shown in Appendix B.

#### Personality Traits Survey

We assessed participants’ personality traits using the Ten Item Personality Inventory (TIPI; Gosling et al., 2003). Each of the 10 items started with “I see myself as” and ended with a personality characteristic (e.g., “I see myself as: Extraverted, enthusiastic”). Participants rated themselves on each item using a 7-point scale (1 = disagree strongly, 7 = agree strongly). For each participant, the survey produced a score for each one of the Big Five personality traits (extraversion, agreeableness, conscientiousness, emotional stability, openness to experience).

## Results

### Factor Analysis of Attitudes

A principal components analysis with varimax rotation was conducted to identify underlying constructs of the 15 items – except for the first item in the survey. There were three factors with an eigenvalue greater than 1. We named factor 1 *Concern with AV* because items (e.g., item 5, items 12–16) loading on it estimated respondents’ level of concern with various types of self-driving vehicles. The factor loading of the items ranged from 0.57 to 0.80. Factor 2 was named *Eagerness to Adopt AV* because items that showed meaningful loading on it (e.g., item 3, 4, 6, and 7, loading range 0.73 –0.85) involved respondents’ likelihood or level of comfort to adopt self-driving technology. Finally, the third factor was named *Willingness to Relinquish Driving Control* given that items (items 2, 9, and 10) loading on it involved respondents’ willingness to ride a car driven by other agents or their perception whether technology is safe and competent enough to take driving control over from a human driver.

The eigenvalues of each factor and factor loadings were summarized in Table 1. Based on these results, using SPSS, we obtained regression factor scores for all three factors and used them as dependent variables in the following analyses.

**Table 1:** Factor loadings and eigenvalues of each factor.

	Factor loading		
Item number	Factor 1 (Concern with AV)	Factor 2 (Eagerness to adopt AV)	Factor 3 (Willingness to relinquish driving control)
2	0.22	0.31	0.55
3	−0.29	0.73	0.41
4	−0.45	0.74	0.25
5	0.57	−0.57	−0.17
6	−0.28	0.85	0.16
7	−0.33	0.84	0.15
8	0.35	0.06	−0.75
9	−0.35	0.41	0.61
10	−0.40	0.44	0.49
11	0.42	−0.27	−0.52
12	0.70	−0.41	0.03
13	0.73	−0.23	−0.25
14	0.79	−0.26	−0.16
15	0.80	−0.24	−0.27
16	0.76	−0.40	−0.27

Eigenvalue	8.15	1.21	1.09

### Analysis of Variance (ANOVA)

A 3-way ANOVA was conducted to compare the effects of different descriptions (a passenger in the front driver seat, a passenger in the rear seat, written description), age groups (younger and middle-aged), and gender for each factor. The two age groups were created based on a median split: 18 – 34 (young adult), 35 + (middle-aged adult). Means and SDs are shown in Table 2.

**Table 2:** Descriptive statistics (mean, SD in parentheses) for attitude factor scores.

	*N*	Concern with AV	Eagerness to adopt AV	Willingness to relinquish control
**Gender**				
Male	161	−0.29 (1.13)	0.13 (1.08)	0.16 (0.93)
Female	256	0.18 (0.86)	−0.08 (0.94)	−0.10 (1.03)
**Age group**				
Young	218	−0.12 (1.08)	0.07 (1.06)	0.06 (0.95)
Middle-aged	196	0.13 (0.89)	−0.07 (0.93)	−0.08 (1.04)
**Introduction type**				
Rear seat	157	0.03 (0.98)	0.03 (1.00)	−0.03 (0.96)
Front seat	145	0.03 (1.00)	0.01 (1.06)	0.07 (1.03)
Text	115	−0.08 (1.02)	−0.06 (0.93)	−0.05 (1.01)

For Concern with AV, there was a significant main effect of gender, *F*(1,402) = 20.21, *p* < 0.001, η^2^ = 0.05, indicating that female respondents reported higher Concern with AV. The main effect of age group on concern was also significant, *F*(1,402) = 6.13, *p* = 0.01, η^2^ = 0.02, indicating that middle-aged respondents reported higher Concern with AV. However, there was no main effect of different introduction (*p* = 0.68) and no interactions among the independent variables (*p* = 0.13 ∼ 0.98).

The main effect of gender on Eagerness to Adopt AV was marginally significant, *F*(1,402) = 3.70, *p* = 0.055, η^2^ = 0.01, suggesting higher Eagerness to Adopt AV in male respondents. The main effects of age group and different introduction were not significant (*p* = 0.09, 0.86). There was no sign of interaction on Eagerness to Adopt AV either (*p* = 0.13 ∼ 0.92).

The main effect of gender on Willingness to Relinquish Driving Control was significant, *F*(1,402) = 6.40, *p* = 0.01, η^2^ = 0.02, supporting higher willingness in male respondents. Neither the main effect of age group nor that of different introduction on willingness was significant (*p* = 0.34, 0.60). There was also no sign of interaction on willingness (*p* = 0.14 ∼ 0.70).

### Regression Analyses

Given that no interactions occurred with the type of introduction to AV, we explored the linear relationships between attitude factors and predictors. A multiple-regression analysis was conducted to explore how personality traits and previous knowledge level would predict the three attitudinal factors toward AV over and above the independent variables (i.e., gender, age, and introduction type). Tests for multicollinearity indicated that there was a very low level of multicollinearity among the predictors (VIF = 1.03 ∼ 1.50). For the previous knowledge level, we generated a dummy variable based on item 1 (whether respondents ever heard of AV or self-driving vehicles before the survey: yes = 1 or no = 0). Interactions among the predictors were not included in the regression models given no evidence of interaction from ANOVAs.

First, the overall model to predict Concern with AV was significant, though accounted for less than 10 percent of the variance, *F*(9,404) = 5.71, *p* < 0.001, *R*^2^ = 0.09. As shown in Table 3, having previous knowledge lessened Concern with AV (β = −0.15, *p* < 0.01), whereas conscientiousness was associated with increased Concern with AV (β = 0.12, *p* = 0.03). Female gender was still associated with greater concern (β = −0.17, *p* < 0.01). Age and emotional stability were marginal predictors of Concern with AV (β = 0.10, −0.11, respectively, *p* = 0.06).

**Table 3:** Multiple regression analysis of the three attitudinal factors toward AV.

Dependent variable	Predictor	β	*t*	*P*^1^
Concern with	Gender	−0.17	−3.36	**<0.01**
AV	Age	0.10	1.94	0.05
	Introduction type	0.04	0.90	0.37
	Previous knowledge	−0.15	−3.00	**<0.01**
	Extraversion	0.02	0.35	0.72
	Agreeableness	0.02	0.31	0.76
	Conscientiousness	0.11	2.18	**0.03**
	Emotional stability	−0.11	−1.94	0.05
	Openness	−0.05	−1.05	0.29

Eagerness	Gender	0.06	1.19	0.24
to adopt AV	Age	−0.04	−0.86	0.39
	Introduction type	0.02	0.44	0.66
	Previous knowledge	0.02	0.35	0.72
	Extraversion	0.04	0.71	0.48
	Agreeableness	−0.06	−1.12	0.27
	Conscientiousness	−0.14	−2.64	**0.01**
	Emotional stability	0.14	2.40	**0.02**
	Openness	0.15	2.81	**<0.01**

Willingness	Gender	0.07	1.28	0.20
to relinquish	Age	0.02	0.29	0.77
driving control	Introduction type	0.01	0.18	0.86
	Previous knowledge	0.20	3.95	**<0.01**
	Extraversion	−0.18	−3.46	**<0.01**
	Agreeableness	−0.05	−0.94	0.35
	Conscientiousness	0.05	0.99	0.32
	Emotional stability	−0.03	−0.50	0.62
	Openness	0.17	3.20	**<0.01**

*Significant values (p < 0.05) shown in bold.*

Second, the overall model to predict Eagerness to Adopt AV was significant, *F*(9,404) = 3.50, *p* < 0.001, *R*^2^ = 0.07. Greater conscientiousness lessened Eagerness to Adopt AV (β = −0.14, *p* = 0.01), while greater emotional stability and openness to experience promoted greater Eagerness to Adopt AV (β = 0.14, 0.15, respectively, *p* < 0.05).

Finally, the model to predict Willingness to Relinquish Driving Control was also significant, *F*(9,404) = 4.99, *p* < 0.001, *R*^2^ = 0.10. Previous knowledge of AV increased Willingness to Relinquish Driving Control (β = 0.20, *p* < 0.01). Increased openness to experience was also positively associated with Willingness to Relinquish Driving Control (β = 0.17, *p* < 0.01), whereas greater extraversion lessened Willingness to Relinquish Driving Control (β = −0.18, *p* < 0.01).

## Discussion

For this younger to middle-aged sample, the framing that AV received through our introductions did not affect attitudinal factors toward AV significantly, nor did framing interact with age and gender to impact the factors. Varying the passenger’s seating position in introductory materials was not found to affect respondents’ attitudes to the extent that the AV ownership vignettes used by Nees (2016) did. This could be because the videos showed safe interaction regardless of passenger seating position. However, consistent with previous findings, we replicated some significant relationships between age and attitudes toward AV as well as gender and attitudes. The age effects from the ANOVA are consistent with the general finding that older individuals are more concerned about AV technology that younger ones, here extending the age effect to a middle-aged sample. The gender effect tended to be significant across all three attitudinal factors, supporting higher Concern with AV, less Eagerness to Adopt AV, and less Willingness to Relinquish Driving Control in women.

The main contribution of our study was in delineating how attitudes toward AV technology cluster into different factors with differing patterns of relationships with age, gender, previous knowledge about AV, and personality. The factor of Concern with AV showed the strongest relationship to age with younger adults less concerned; to gender, with men less concerned; and personality traits, with conscientious people more concerned. These age and gender findings are consistent with evidence from previous survey studies in this area (e.g., Payre et al., 2014; Kyriakidis et al., 2015), and the personality-related findings extend the literature on how different personality traits relate to AV-related attitudes. The respondents who had prior knowledge of AV also showed less concern with AV. Souders et al. (2017) found that greater familiarity with certain advanced driver assistance systems (lane departure warning, adaptive cruise control, and emergency braking systems) was predictive of willingness to use these systems.

Combined, these findings suggest that even limited exposure to advanced vehicle technologies is beneficial toward either allaying concerns (as the current study found with AV), and/or increasing usage intentions. Increasing familiarity with and/or exposure to these technologies seems like one of the best ways to reduce concerns and increase positive attitudes toward vehicle automation. Indeed, a deployment of a low-speed driverless shuttle in Minnesota in early 2018 found that 84% of passengers were apprehensive about AV prior to their ride, but 95% of passengers reported feeling safe during their experience (KSTP, 2018).

The factor termed Eagerness to Adopt AV showed a mix of relationships with personality traits. Those higher in conscientiousness were less eager to adopt AV, those higher in openness to experience and with greater emotional stability were more eager. Conscientiousness is related to a need for personal achievement, order, and persistence (Costa et al., 1991), and individuals high in conscientiousness are more likely to be conformists (DeYoung et al., 2002). Regarding technology acceptance, conscientiousness has been found to moderate the relationship between subjective norms and the intention to use a technology (Devaraj et al., 2008). In this light, it makes sense that individuals higher in conscientiousness show more reluctance to adopt a new and largely unproven AV technology that replaces their performance in the driving task (though it is noteworthy that conscientiousness was not a significant negative predictor of the Willingness to Relinquish Driving Control factor). Openness to experience has been found to be positively correlated to sensation seeking (Roberti, 2004), which itself has been found to be related to intention to use AVs (Payre et al., 2014). In addition to this, Devaraj et al. (2008) found that neuroticism (i.e., low levels of emotional stability) was negatively associated with the perceived usefulness of a new technology. Hence, the current study’s findings related to the Eagerness to Adopt AV factor are consistent with previous work.

The third factor, Willingness to Relinquish Driving Control, also showed a mix of relationships. Those who had prior knowledge of AV were more inclined to give up control. Those higher in openness to experience were also more willing to relinquish driving control, whereas those higher on extraversion were less willing to abandon personal driving. As stated earlier, prior knowledge of AV was related to the Concern with AV factor, and it follows that prior knowledge might also be related to a greater Willingness to Relinquish Driving Control. Similarly, the positive relationship between openness to experience and the Eagerness to Adopt AV might also be in line with that between openness to experience and the Willingness to Relinquish Driving Control. However, it is unclear why extraversion was negatively associated with Willingness to Relinquish Driving Control. To further clarify possible mechanisms underlying the mix of associations, investigators need to see if the associations between personality traits and these attitudinal factors toward AV are replicated elsewhere.

As mentioned in the introduction, many researchers have argued that AV technology may hold the most benefit for older adults who can no longer drive safely. If AVs eventually drive better than the average driver and if older drivers can be encouraged to shift to AVs, given their greater vulnerability in traffic crashes, hypothesized age-related benefits may be realized. Nonetheless, in the United States, middle-aged and above drivers, those age 35–74 in our study, comprise 47% of the driving population, and numerically, suffered the greatest absolute burden from crash deaths, as seen in Table 4. It is also worth noting that the middle-aged respondents in our sample are likely approaching or at their peak driving ability, which could affect how useful they perceive AVs to be in their everyday lives.

**Table 4:** Traffic crash deaths in the United States in 2016 by age group.

Age group	Population size	Crash deaths
<13	52,771,635	1,023
13–15	12,400,425	407
16–19	16,933,008	2,413
20–24	22,381,028	4,379
25–29	22,890,884	3,789
30–34	21,786,359	3,102
35–39	20,773,905	2,565
40–44	19,696,251	2,420
45–49	20,947,623	2,463
50–54	21,839,056	2,854
55–59	21,980,108	2,774
60–64	19,483,036	2,389
65–69	16,820,083	1,972
70–74	11,810,247	1,440
75–79	8,367,895	1,188
80–84	5,865,639	1,052
85+	6,380,331	1,112
Total	323,127,513	37,461

*Data from http://www.iihs.org/iihs/topics/t/general-statistics/fatalityfacts/overview-of-fatality-facts/2016#Age-and-gender, accessed 6/15/2018*.

In 2016, 13,683 drivers age 16–34 were killed in vehicle crashes, whereas 18,877 drivers age 35 to 74 died. People age 65+ comprised 15% of the United States population and suffered 6,764 crash deaths. Hence the greatest societal benefits may accrue if the middle-aged population studied here can be convinced to leave driving up to an AV. As our results indicate, they may need more convincing to allay concerns. Particularly for Willingness to Relinquish Driving Control where age was not a significant factor, having more information about the safety of AV technology will be essential, though such information is difficult to get now given proprietary interests involved in AV technology development. Also, given the varying relationships to personality, particularly to extraversion, different information campaigns may be needed to persuade people to relinquish personal driving. A three-tiered approach seems warranted: (1) providing accurate, publicly verifiable information about safety to address such concerns, (2) providing information relevant to encouraging adoption stressing benefits (Melenhorst et al., 2006) for older drivers, and (3) providing arguments in favor of being driven, such as freedom to pursue social interests, such as safe social media use, for extraverts.

There are several caveats to these conclusions. First, the MTurk population is not representative of the general population, particularly for age, skewing too young, too female, and too educated, though with lower than average incomes (Paolacci et al., 2010). In particular, the lack of older adults (e.g., age 65+) in our sample did not allow us to include a separate age group for older adults in ANOVA analyses and it might be one possible reason why an age effect was not prominent across our analyses. In 2018 in the United States, about a third of those age 65+ years do not use the Internet^4^ hence their opinions are unlikely to be well represented here, even by the relatively few seniors who participate as MTurk workers. Further, MTurk workers, who register and provide labor for this service online, are perhaps more likely to embrace technology than the general population and hence show more favorable attitudes to AV, so we may be overestimating levels of acceptance. It is also worth noting that the data for this study was collected before 2018, where there has been a rash of negative press surrounding AV, including the first pedestrian fatality. Future studies should investigate whether observed relationships in the current study involving prior knowledge of AV persist despite recent negative press. It is also worth noting that the source of prior knowledge might play a role in forming less concern with AV technology. Specifically, making decisions based on actual experience can make people underweight the probability of rare events (Hertwig et al., 2004). Similarly, having actual prior experience with advanced driver assistance systems may decrease the perceived probability of an autonomous vehicle malfunction or accident, while getting only the description of AV technology (e.g., via media source) may increase the perceived probability of such events. However, here prior knowledge was a dichotomized variable, thus it did not allow us to track how the participants obtained prior knowledge of AV as well as differences in extent of knowledge. Future studies should investigate these relationships.

In sum, the current study examined how different types of introduction to AV technology influenced attitudes toward the new technology and how individual difference variables predicted the attitudes. We first captured different attitudinal factors toward AV technology, which allowed us to describe different roles of age, gender, previous knowledge level, and personality traits in predicting these attitudes. Our results suggest that information dissemination campaigns need to be sensitive to these differing attitudinal factors if middle-aged consumers are to be persuaded to adopt AV technology.

## Ethics Statement

This study was carried out in accordance with the recommendations of the Institutional Review Board, Human Subjects Committee at Florida State University with typed informed consent from all participants. All participants gave written informed consent in accordance with the Belmont Report. The protocol was approved by the Florida State University IRB.

## Author Contributions

NC, CS, and CY developed the idea for the study. CS and CY prepared the materials and collected the data. NC and JY conducted the statistical analyses. JY, NC, DS, and CS drafted the manuscript.

## Conflict of Interest Statement

The authors declare that the research was conducted in the absence of any commercial or financial relationships that could be construed as a potential conflict of interest.

## Footnotes

1 https://www.bts.gov/statistical-products/surveys/national-household-travel-survey-daily-travel-quick-facts

2 https://osf.io/9at7m/

3 https://osf.io/9at7m/

4 http://www.pewinternet.org/chart/internet-use-by-age/, accessed 6/15/2018

## Appendix A

### Written Description of AV

Autonomous vehicles, including the self-driving car, have recently been emerging to the mainstream of modern technology. The United States Department of Transportation’s National Highway Traffic Safety Administration (NHTSA) defines the autonomy of a vehicle in terms of five distinct levels with “Level 0” as no-automation, the driver has sole control and responsibility over the vehicle at all times, and “Level 4” as a fully autonomous vehicle, the vehicle is responsible for all functions and monitoring the environment. In a Level 4 vehicle, the driver is not expected to assume any intervention with driving or controls and may be seated throughout the journey wherever he or she pleases. The only use of the driver would be for navigation or destination input. In fact, a Level 4 vehicle could drive with or without passengers.

The collection of abilities a Level 4 autonomous vehicle has is remarkable to say the least. For instance, one feature of a vehicle could be automatically adaptive cruise control based on speed and distance; the vehicle would be able to determine on its own a rate of speed at any given time by utilizing the brakes and gas autonomously depending on the distance and speed of the vehicles around it. Another feature would be lane changing; again, the vehicle would determine the speeds and distances of vehicles and other objects around it in order to safely turn into different lanes on the road. Other features include, but are not limited to, entering and exiting highways, adapting to weather and road conditions, and collision prevention.

As mentioned previously, neither a driver nor passenger is necessary for a Level 4 autonomous vehicle. The vehicle would have full responsibility in not only all controls and functions, but also monitoring the roadway and the environment. Considering the roadways will mostly be filled with vehicles of much lower levels of automation, the autonomous vehicle will not be able to communicate with these other vehicles and will depend on its own constant awareness of speed and distance measurements of surrounding vehicles and other objects to maneuver around them while driving in order to avoid collisions. The United States Department of Transportation’s NHTSA ensures responsibility for safety regulation and enforcement of all autonomous vehicles.

## Appendix B

### Survey Items

(1) Had you ever heard of autonomous and/or self-driving vehicles before participating in this survey?

• Yes
• No

On a scale of 1–10, with 1 being **“highly unlikely”** and 10 being **“highly likely,”** please answer the following questions:

(2) What is the likelihood that you would ride as a passenger in a car driven by a taxi driver, assuming that it would be at no additional cost over other driving options including driving a personally owned vehicle?

**Table:**

1	2	3	4	5	6	7	8	9	10
Highly Unlikely				Neutral					Highly Likely

(3) What is the likelihood that you would ride as a passenger in an autonomous or self-driving vehicle, assuming that it would be at no additional cost over other driving options including driving a personally owned vehicle?

**Table:**

1	2	3	4	5	6	7	8	9	10
Highly Unlikely				Neutral					Highly Likely

Please give us your opinion in terms of your agreement for the following statements:

(4) “I would be comfortable with driving or riding in an autonomous or self-driving vehicle.”

• Strongly agree
• Agree
• Neither agree nor disagree
• Disagree
• Strongly disagree

(5) “I would be concerned about driving or riding in an autonomous or self-driving technology.”

• Strongly agree
• Agree
• Neither agree nor disagree
• Disagree
• Strongly disagree

(6) “If self-driving technology were now available as optional equipment on my next car purchase, I would buy or lease this technology.”

• Strongly agree
• Agree
• Neither agree nor disagree
• Disagree
• Strongly disagree

(7) “I would buy or lease a completely autonomous vehicle (Level 4) if one were available.”

• Strongly agree
• Agree
• Neither agree nor disagree
• Disagree
• Strongly disagree

(8) “I think that autonomous vehicles can never be safer than those driven by humans.”

• Strongly agree
• Agree
• Neither agree nor disagree
• Disagree
• Strongly disagree

(9) “I think advances in science and technology will allow driverless cars to be as safe as human drivers.”

• Strongly agree
• Agree
• Neither agree nor disagree
• Disagree
• Strongly disagree

(10) “Computers are capable of the same quality of decision making as human drivers.”

• Strongly agree
• Agree
• Neither agree nor disagree
• Disagree
• Strongly disagree

(11) “I can drive better than a computer.”

• Strongly agree
• Agree
• Neither agree nor disagree
• Disagree
• Strongly disagree

How concerned are you about the following possible scenarios with **completely self-driving vehicles (Level 4)?**

(12) I would be concerned with riding in a vehicle with no driver controls available (no steering wheel, no brake pedal, and no gas pedal/accelerator):

• Strongly agree
• Agree
• Neither agree nor disagree
• Disagree
• Strongly disagree

(13) I would be concerned with self-driving vehicles moving by themselves from one location to another while unoccupied:

• Strongly agree
• Agree
• Neither agree nor disagree
• Disagree
• Strongly disagree

(14) I would be concerned with commercial vehicles such as heavy trucks or semi-trailer trucks that are completely self-driving:

• Strongly agree
• Agree
• Neither agree nor disagree
• Disagree
• Strongly disagree

(15) I would be concerned with public transportation such as busses that are completely self-driving:

• Strongly agree
• Agree
• Neither agree nor disagree
• Disagree
• Strongly disagree

(16) I would be concerned with taxis that are completely self-driving:

• Strongly agree
• Agree
• Neither agree nor disagree
• Disagree
• Strongly disagree
